# Effect of Physical Activity on Plasma PCSK9 in Subjects With High Risk for Type 2 Diabetes

**DOI:** 10.3389/fphys.2019.00456

**Published:** 2019-04-30

**Authors:** Kari Antero Mäkelä, Juhani Leppäluoto, Jari Jokelainen, Timo Jämsä, Sirkka Keinänen-Kiukaanniemi, Karl-Heinz Herzig

**Affiliations:** ^1^Research Unit of Biomedicine, Faculty of Medicine, University of Oulu, Oulu, Finland; ^2^Center for Life Course Health Research, Faculty of Medicine, University of Oulu, Oulu, Finland; ^3^Medical Research Center Oulu, Oulu University Hospital, University of Oulu, Oulu, Finland; ^4^Research Unit of Medical Imaging, Physics and Technology, University of Oulu, Oulu, Finland; ^5^Department of Diagnostic Radiology, Oulu University Hospital, Oulu, Finland; ^6^Health Center of Oulu, Oulu, Finland; ^7^Healthcare and Social Services of Selänne, Pyhäjärvi, Finland; ^8^Biocenter Oulu, University of Oulu, Oulu, Finland; ^9^Department of Gastroenterology and Metabolism, Poznan University of Medical Sciences, Poznań, Poland

**Keywords:** LDL cholesterol, PCSK9, physical activity, prediabetes, type 2 diabetes

## Abstract

**Background:**

Proprotein convertase subtilisin/kexin type 9 (PCSK9) is a liver serine protease regulating LDL cholesterol metabolism. PCSK9 binds to LDL receptors and guides them to lysosomes for degradation, thus increasing the amount of circulating LDL cholesterol. The aim of the study was to investigate associations between physical activity and plasma PCSK9 in subjects with high risk for type 2 diabetes (T2D).

**Methods:**

Sixty-eight subjects from both genders with a high risk for T2D were included to a randomized controlled trial with a 3-month physical activity intervention. Physical activity intensities and frequencies were monitored throughout the intervention using a hip worn portable accelerometer. The plasma was collected before and after intervention for analysis of PCSK9 and cardiovascular biomarkers.

**Results:**

Plasma PCSK9 did not relate to physical activity although number of steps were 46% higher in the intervention group than in the control group (*p* < 0.029). Total cholesterol was positively correlated with plasma PCSK9 (*R* = 0.320, *p* = 0.008), while maximal oxygen uptake was negatively associated (*R* = -0.252, *p* = 0.044). After the physical activity intervention PCSK9 levels were even stronger inversely associated with maximal oxygen uptake (*R* = -0.410, *p* = 0.0008) and positively correlated with HDL cholesterol (*R* = 0.264, *p* = 0.030). Interestingly, plasma PCSK9 levels were higher in the beginning than at the end of the study.

**Conclusion:**

The low physical activity that our subjects with high risk for T2D could perform did not influence plasma PCSK9 levels. Intervention with higher physical activities might be more effective in influencing PCSK9 levels.

## Introduction

High circulating LDL (low density lipoprotein) cholesterol concentration is the most important cardiovascular risk factor. Type 2 diabetes (T2D) is known to promote the production of LDL cholesterol potentiating the cardiovascular risks. Thus, pharmacological treatments with statins has been widely used to reduce LDL cholesterol (Momtazi et al., 2017).

Proprotein convertase subtilisin/kexin type 9 (PCSK9) is a serine protease which binds to LDL and other lipoprotein receptors convoying the complex to intracellular degradation compartments (Seidah et al., 2014). The enzyme is abundantly expressed in liver hepatocytes and circulates in plasma. The physiological role of PCSK9 is to promote the degradation of LDL receptors and thereby to regulate the amount of circulating LDL cholesterol. Gain-of-function mutations of PCSK9 cause severe hypercholesterolemia, whereas loss-of-function types lead to decreased circulating LDL cholesterol levels, protecting from cardiovascular disease (CVD) (Cohen et al., 2005). Monoclonal PCSK9 antibodies are already on the market and small interfering RNAs studied as potentially effective drugs against hypercholesterolemia (Lloyd-Jones et al., 2017; Momtazi et al., 2017; Stoekenbroek et al., 2018).

Associations between plasma PCSK9 and CVD risk variables have been investigated in healthy subjects and significant correlations were observed for BMI, LDL cholesterol, triglycerides, insulin and glucose (Lakoski et al., 2010). In diabetic subjects plasma PCSK9 levels were higher than in non-diabetic subjects (Cariou et al., 2010; Nekaies et al., 2015; Ibarretxe et al., 2016). Contrary to these reports, no significant differences in plasma PCSK9 levels were observed in a cohort of subjects with normal glucose metabolism, impaired glucose metabolism and T2D (Brouwers et al., 2011) or in non-diabetic and T2D subjects (Vergès et al., 2011).

Importantly, statin therapy is known to raise plasma PCSK9 levels and the increase appears to be mediated by transcriptional activation of sterol response element binding protein 2 (SREBP2) (Horton et al., 2003; Careskey et al., 2008; Mayne et al., 2008). Statins induce PCSK9 gene expression via hepatocyte nuclear factor 1 alpha (HNF1a) and reduce LDL cholesterol in dyslipidemia hamsters (Dong et al., 2010).

Only few investigations have examined the associations between PCSK9 concentrations and lifestyle factors. The results, however, are confusing. PCSK9 levels have been reported to be either decreased, increased or not affected at all to the physical exercises (Arsenault et al., 2014; Kamani et al., 2015; Sponder et al., 2017).

We have earlier demonstrated inverse associations between objectively measured physical activity (PA) and glucose and lipid metabolism in subjects with high risk of developing T2D (Herzig et al., 2014). Due to the conflicting results on the effects of physical activity on PCSK9 levels in the literature, we analyzed the plasma PCSK9 concentrations in these subjects and evaluated the associations between PCSK9 before and after a 3-month PA intervention. We also investigated the effects of physical activity in statin treated and untreated subjects.

## Materials and Methods

Our study included 68 sedentary, and prediabetic subjects who participated in the randomized controlled trial (RCT) for 3 months as described previously (Herzig et al., 2014). The subjects were divided into intervention (*n* = 33) and control group (*n* = 35). Ages and body weights in the intervention group were 58.1 ± 9.9 years and 92.4 ± 19.4 kg, and 16 of them used statins. The consecutive values for control group were 59.5 ± 10.8 years and 84.6 ± 14.4 kg, and 14 of them used statins. All subjects completed the FINDRISC questionnaire for T2D^1^. Those with the score >15 were further evaluated by an oral glucose tolerance test. These tests were done before the start of the study and 48–72 h after the last exercise. The subjects met the WHO criteria^2^ for impaired fasting glucose (≥ 5.6 and < 7.0 and 2 h glucose < 7.8 mmol l^-1^) or impaired glucose tolerance (fasting glucose < 7.8 and 2 h glucose ≥ 7.8 and < 11.1 mmol l^-1^). The trial was also registered under *ClinicalTrials.gov* identifier NCT01649219. All subjects continued their regular medication during the study. Before the intervention all subjects received via a common lecture information on the importance of regular PA and weight reduction to prevent the development of T2D as best standard care. The control group did not receive supervised exercise and no placebo was given.

The intervention group had supervised 60 min sessions three times a week. Each session started with a 5 min warm-up with stretching followed by a 20 min walk at a speed of ∼3–4 km h^-1^. The session continued with a 5 min stretching and balance training followed by a 20 min walk and finished with a 10 min stretching and balance exercise. After 1.5 months the walking time was increased to 45 min by eliminating stretching and balance training between the walking periods. The goal of the intervention group was to reach the current physical activity guidelines for adults and T2D subjects being at least 150 min moderately vigorous physical activity weekly (Garber et al., 2011). All subjects carried accelerometer on their belt close to the iliac crest (Newtest Exercise Monitor, Newtest, Oulu, Finland) during waking hours except during aquatic activities every day for 3 months. Our accelerometers were validated as follows: the number of steps registered by the accelerometer were checked on the force plate with identical results. Weighted averages of accelerations in walking/running speeds of 3, 6, and 9 km h^-1^ accounted for 92% of the variance in the energy expenditure. The median whole-day (14 h) wear time for the accelerometer was 78 days (Herzig et al., 2014). The mean number of daily steps was 5870 ± 3277 in the intervention group and 4034 ± 3460 in the control group at the low acceleration levels (*p* < 0.029). The subjects were overweight or obese and their maximal oxygen uptake capacity was low (Herzig et al., 2014). Thus, they were unable to meet physical activity guidelines (Garber et al., 2011). It should be noted that less than 5 % of the health adult subjects meet the physical activity guidelines (Troiano et al., 2008).

The mean outdoor temperatures during these months varied in the city of Oulu (65° latitude) from -13.9°C to 1.5°C (Finnish Meteorological Institute). Daylight hours increased from around 4 to around 13.5 h from January to April. Two blood samples were taken for the study: the first one in January and the second in April. The samples were taken in the morning between 8 and 10 am.

The specificity of the PCSK9 –antibody used in the commercial ELISA PCSK9 –kit was tested prior to ELISA measurements. Two EDTA-plasma samples (35 μl from both subjects containing 424.3 and 113.2 ng/ml PCSK9 as measured afterward with PCSK9 ELISA) were used for immunoprecipitation (Pierce Direct IP Kit, ThermoFisher Scientific, Rockford, IL, United States). In short, PCSK9 antibody, (Catalog # AF3888, R&D SYSTEMS^TM^, Oxon, United Kingdom) was coupled covalently to amino-reactive resin. Eluted samples were run on SDS–PAGE gel (4 % stacking gel; 12 % running gel) and immunoblotted with PCSK9 antibody to identify the antigen from the sample. PCSK9 exists in several forms (Naureckiene et al., 2003; Seidah et al., 2014). The antibody detected a 74 kDa PCSK9 precursor protein, as well as 60 and 14 kDa cleavage products (Supplementary Material). Secondary antibody itself stained only weakly a protein near 60 kDa and thus did not significantly interfere with the recognition of the antigens. The antibody tested here was the same used in PCSK9 ELISA–kit. Thus, the antibody used in our assay was specific to PCSK9.

The plasma samples were analyzed using PCSK9 ELISA–kits (Human Proprotein Convertase 9/PCSK9, R&D SYSTEMS^TM^, Oxon, United Kingdom). The reported intra-assay coefficients of variations (%CV) for three test samples were between 4.1 and 6.5 %, and the inter-assay variations were between 4.1 and 5.9 %.

### Statistical Analyses

Data were analyzed using the SPSS statistical package (PASW statistics 18 for windows, SPSS inc., Chicago, IL, United States). Wilcoxon signed rank test was used to calculate changes in plasma PCSK9 between untreated and statin treated subjects or between basal and 3 months. Spearman correlations between basal PCSK9 and its changes during 3 months and physical activity (daily steps) or clinical parameters were calculated by univariate correlation analysis. The quantitative changes in PCSK9 and study variables were calculated by repeated measures ANOVA with fixed effects. In figures and text means and SDs are given. Statistical significance is indicated by ^∗^*p* < 0.05.

## Results

Plasma levels of PCSK9 were correlated at baseline with several clinical parameters in a univariate analysis (Table 1). Total cholesterol was positively (*R* = 0.320, *p* = 0.008) and maximal oxygen uptake inversely associated with PCSK9 levels (*R* = -0.252, *p* = 0.044). After the 3-month intervention significant correlations were observed for changes in maximal oxygen uptake (*R* = -0.410, *p* = 0.0008) and HDL cholesterol (0.264, *p* = 0.030). We did not observe significant correlation between physical activity (daily steps) and plasma PCSK9 levels (Table 1).

**Table 1:** Correlations between PCSK9 and clinical parameters at baseline and after the 3 months intervention in subjects with high T2D risk (*n* = 61–68).

Variable	R	*P*-value	R	*P*-value
			
	Baseline		Difference	
Age	0.050	0.684		
Fasting insulin	-0.134	0.274	-0.094	0.445
2-h Insulin	-0.196	0.110	-0.00098	0.994
Fasting glucose	-0.197	0.108	0.197	0.108
2-h glucose	-0.072	0.561	-0.012	0.925
HOMA	-0.127	0.303	0.172	0.160
Total cholesterol	**0.320**	**0.008^∗∗^**	-0.082	0.505
LDL cholesterol	0.153	0.212	-0.134	0.275
HDL cholesterol	0.226	0.064	**0.264**	**0.030^∗^**
Triglycerides	0.198	0.105	0.039	0.751
Systolic BP	-0.128	0.301	-0.093	0.477
Diastolic BP	0.026	0.837	0.085	0.514
Body weight	0.040	0.748	-0.057	0.648
Maximal VO_2_	-**0.252**	**0.044^∗^**	-**0.410**	**0.0008^∗∗∗^**
Physical activity#			-0.040	0.750


*^∗^ ≤ 0.05; ^∗∗^ ≤ 0.01; and ^∗∗∗^ ≤ 0.001. HOMA, Homeostasis model assessment; LDL, low density lipoprotein; HDL, high density lipoprotein; BP, blood pressure; VO_*2*_, O_*2*_ volume. #Average daily physical activity during 3 months. The bolding highlights the statistical significances.*

**FIGURE 1:**
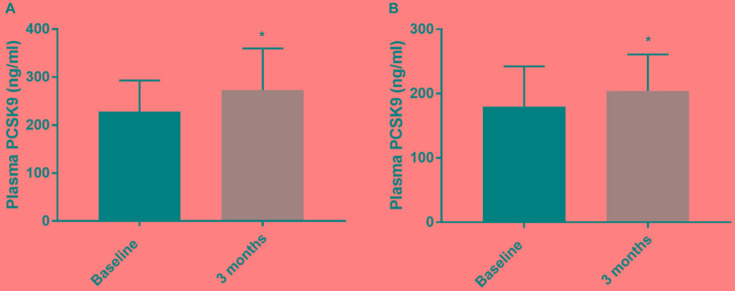
Plasma PCSK9 levels (ng/ml) in prediabetic subjects under statin therapy **(A)** and without statins **(B)** at baseline and at 3 months. Mean ± SD is given. The groups were significantly different from each other (Wilcoxon signed rank test; *p* < 0.0145 for statin treated subjects and *p* < 0.0160 for subjects without statin therapy). ^∗^ ≤ 0.05.

**FIGURE 2:**
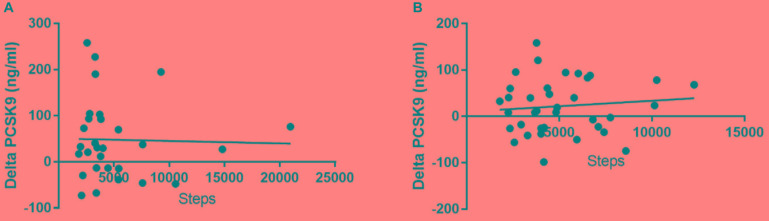
Plasma PCSK9 levels did not correlate significantly to steps (i.e., physical activity) in subjects under statin **(A)** or without statin **(B)** (R square = 0.0006776 and 0.01231, respectively).

Since statin therapy is known to rise plasma PCSK9 levels (Careskey et al., 2008; Mayne et al., 2008), we evaluated the associations between PCSK9 with or without statin therapy and daily physical activity. After the 3-month intervention PCSK9 levels were significantly higher than those at the baseline (Figure 1A,B; Wilcoxon signed rank test; *p* < 0.0145; *p* < 0.0160). We also correlated changes in plasma PCSK9 to physical activity (daily steps), but no significant correlations were observed in subjects with or without statin (Figure 2A,B).

**FIGURE 3:**
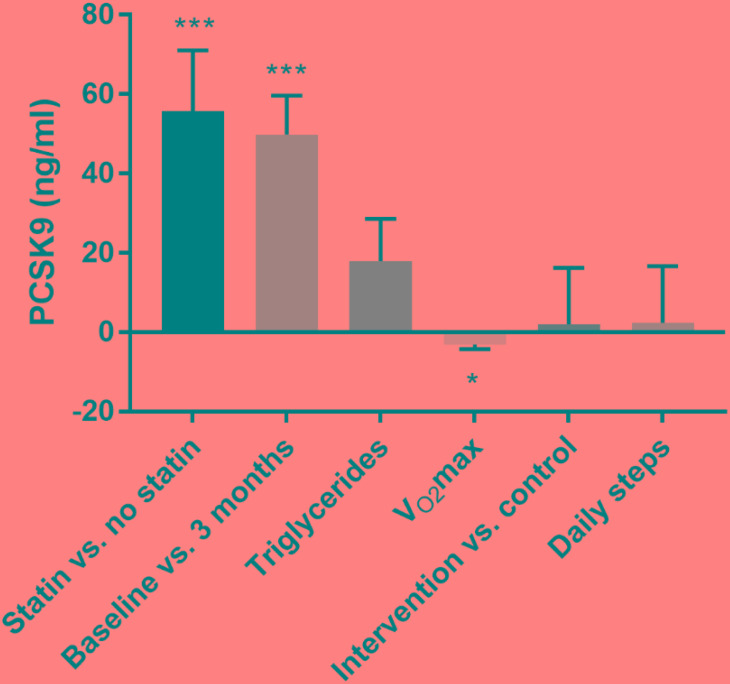
Repeated measures ANOVA using fixed effects between quantitative effects in plasma PCSK9 and dependent variables. Mean ± SD is given. ^∗^ ≤ 0.05 and ^∗∗∗^ ≤ 0.001.

To estimate the quantitative effects of the different variables on the observed increase in plasma PCSK9 we carried out repeated measures analysis. Significant effects were observed between the duration of the intervention and maximal oxygen uptake. The duration of the intervention showed significant effects of 49.8 μg/l higher PCSK9 plasma levels at 3 months than at baseline (Figure 3; *p* < 0.0001). Statin treated subjects had 55.7 μg/l higher plasma PCSK9 levels than untreated subjects (*p* = 0.0006). Increase in oxygen uptake of 1 ml min^-1^kg^-1^ responded to decrease of 3.1 μg/l in plasma PCSK9 (*p* = 0.0139) and increase of 1 mmol/l in triglycerides responded to increase of 17.9 μg/l in plasma PCSK9 (not significant). No significant effects were found with PCSK9 and other variables presented in Figure 3 (intervention vs. control: 2.05 ± 14.24 μg/l; daily steps: 2.4 ± 14.2 μg/l).

## Discussion

In the present study we did not find a significant correlation between plasma PCSK9 levels and daily physical activity in our subjects with high risk for T2D. No association between PCSK9 and glucose markers was observed in the whole study group.

Effects of physical activity on plasma PCSK9 levels have been investigated previously in other studies. Obese and dyslipidemic men participated in a 1-year lifestyle program (Arsenault et al., 2014). PCSK9 levels did not correlate with physical exercise consisting of moderate aerobic activity for 160 min/week and increased occupational physical activity. Kamani et al. (2015) observed that PCSK9 levels decreased at 3 months and did not change at 6 months in healthy hospital employees using stairs instead of elevators. However, the maximal oxygen uptake was improved both at 3 and 6 months, which is in line with our study. Recently, Sponder et al. (2017, 2018) studied PCSK9 levels in 109 men with cardiometabolic risk factors participating in endurance training for 8 months and found that PCSK9 levels were increased at 2, 6, and 8 months. The reason for the increased PCSK9 levels is unclear since no control subjects were used in the study (Sponder et al., 2017). In a follow up study with the same subjects, the authors reported an increase in sRAGE (soluble receptor of advanced glycation end products) levels (Sponder et al., 2018) which might be due to the increased physical activity or other confounding factors. As the study group and the samples were similar in both studies (Sponder et al., 2017, 2018) it is possible that the observed increase in PCSK9 could have been affected by the same factors.

A previous study (Arsenault et al., 2014) and our present that physical activity interventions do not affect plasma PCSK9 levels in obese and sedentary subjects, but a decrease (Kamani et al., 2015) or increase (Sponder et al., 2017) in plasma PCSK9 were observed in subjects after structured exercises. The controversial results from the earlier reports evidently derive from different physical activity exposures and lack of objective physical activity measurement methods.

Previously, statins have been shown to elevate plasma PCSK9 (Horton et al., 2003; Careskey et al., 2008; Mayne et al., 2008; Nozue et al., 2013). We found similar results but after the 3 months PCSK9 levels were significantly increased also in untreated subjects. This is an unexpected finding, since in an earlier study plasma PCSK9 levels were decreased after a 3 month exercise intervention (Kamani et al., 2015). Methodological factors such as timing of blood sampling and the PCSK9 assay do not explain this discrepancy. Plasma PCSK9 presents a diurnal rhythm showing decreasing levels in early evening and a 3–4-fold increase at late night (Persson et al., 2010; Chen et al., 2014). The blood samples in our and Kamani et al.’s (2015) study were taken in the morning when plasma PCSK9 levels were relatively stable and the group used the PCSK9 ELISA from the same vendor.

In order to estimate quantitative effects of dependent variables on the observed increase in plasma PCSK9 we carried out repeated measures analysis between changes in PCSK9 and dependent variables during the 3-month intervention. Significant effects were observed between PCSK9 and duration of the intervention (baseline vs. 3 months), statin therapy, and maximal oxygen uptake but not with triglycerides or whole day physical activity. In our study during the 3-month intervention there were notably changes in environmental factors. Changes in respective daylight durations were from around 4 h in the beginning of the January to about 13.5 h at the end of the March. In a study by Sponder et al. (2017) a significant increase in PCSK9 levels was observed between the baseline and 8 months of training, but the sampling time of the season was not given. In addition to possible seasonal changes, other unknown confounding factors might be the reason for the PCSK9 increase.

Our study has strengths but also limitations. The strength of the present study was the RCT in a high risk group for T2D, designed to minimize effects of confounding factors. Furthermore, we used validated accelerometers for the first time in PCSK9 studies to register the daily physical activities for 3 months. As weakness, we had only a small number of subjects. In addition, a longer duration of our study could have led to significant changes PCSK9 levels.

In the present study plasma PCSK9 did not significantly associate to physical activity during the 3 months of intervention in our subjects. They had a high risk for T2D, were overweight and sedentary and therefore able to perform only light physical activity that may have affected our results. Further studies with moderate or moderately vigorous physical activities are required.

## Ethics Statement

The study protocol was approved by the institutional ethics committee (Northern Ostrobothnia Hospital District). All subjects gave their informed written consent. The Municipal Board of the Northern Ostrobothnia Hospital District approved the trial under the registration number 113/2009. The trial was also registered under NCT01649219 (clinicaltrials.gov). The data obtained for the current study was produced after the original trial.

## Author Contributions

K-HH, JL, SK-K, TJ, and KM designed the study and contributed to the data analysis. KM performed the optimization for immunoprecipitation, western blotting protocols, and analyzed the ELISA assays data and drafted the manuscript. JJ performed the statistical analysis. All authors contributed to the writing of the manuscript.

## Conflict of Interest Statement

The authors declare that the research was conducted in the absence of any commercial or financial relationships that could be construed as a potential conflict of interest.

## Footnotes

1 https://www.idf.org/type-2-diabetes-risk-assessment/

2 https://www.who.int/diabetes/action_online/basics/en/index2.html
