# Supplementary material for: Effect of Physical Activity on Plasma PCSK9 in Subjects With High Risk for Type 2 Diabetes
Source: Front Physiol. 2019 Apr 30;10:456. doi: 10.3389/fphys.2019.00456 (PMC6502968; doi:10.3389/fphys.2019.00456)
Supplement: Supplementary file 1 [file Image_1.pdf]

Supplementary material

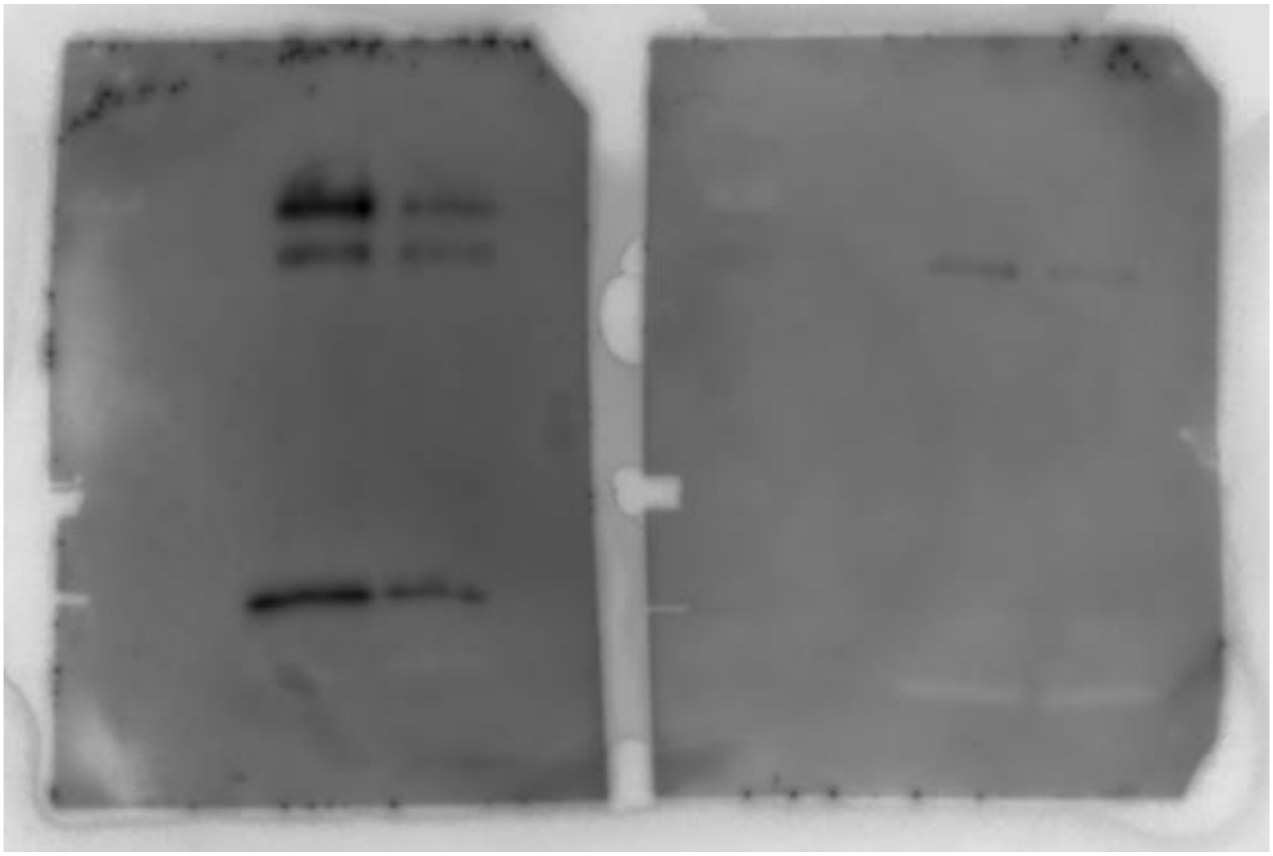

Figure 1. PCSK9 antibody was coupled covalently to amino-reactive resin. Two 35 µl human plasma samples containing 424.3 and 113.2 ng/ml (from left to right) PCSK9 (as measured with PCSK9 ELISA) were used for immunoprecipitation. Eluted samples were run on SDS-PAGE gel and immunoblotted with PCSK9 antibody to identify the antigen from the sample. The antibody detected a 74 kDa PCSK9 precursor protein and 60 kDa as well as 14 kDa cleavage products (left blot). Secondary antibody itself did not recognize these antigens (right blot).
